# The ABO Blood Group is an Independent Prognostic Factor in Patients With Resected Non-small Cell Lung Cancer

**DOI:** 10.2188/jea.JE20140102

**Published:** 2015-02-05

**Authors:** Koichi Fukumoto, Tetsuo Taniguchi, Noriyasu Usami, Koji Kawaguchi, Takayuki Fukui, Futoshi Ishiguro, Shota Nakamura, Kohei Yokoi

**Affiliations:** Department of Thoracic Surgery, Nagoya University Graduate School of Medicine, Nagoya, Japan; 名古屋大学大学院医学系研究科病態外科学講座 呼吸器外科学

**Keywords:** non-small cell lung cancer, ABO blood group, surgery, prognostic factor

## Abstract

**Background:**

The ABO blood group is reported to be associated with the incidence and patient survival for several types of malignancies. We conducted a retrospective study to evaluate the prognostic significance of the ABO blood group in patients with resected non-small cell lung cancer (NSCLC).

**Methods:**

A total of 333 patients (218 men and 115 women) with resected NSCLC were included in this study. In addition to age, sex, smoking status, preoperative serum carcinoembryonic antigen (CEA) level, operative procedure, histology of tumors, pathological stage (p-stage), and adjuvant therapy, the association between the ABO blood group and survival was explored.

**Results:**

The 5-year overall and disease-free survival rates were 83.0% and 71.6% for blood group O, 67.2% and 62.3% for blood group A, 68.8% and 68.8% for blood group B and 69.2% and 65.3% for blood group AB, respectively. A multivariate analysis for overall survival showed the ABO blood group (group A vs. group O: HR 2.47, group AB vs. group O: HR 3.62) to be an independent significant prognostic factor, in addition to age, sex, smoking status, p-stage, and serum CEA level. A multivariate analysis for disease-free survival also showed the ABO blood group to be an independent significant prognostic factor.

**Conclusions:**

The ABO blood group is an independent prognostic factor in patients with resected NSCLC. Studies of other larger cohorts are therefore needed to confirm the relationship between the ABO blood group and the prognosis among patients with resected NSCLC.

## Abstract (ja)

**【背景】:**

ABO血液型はいくつかのがん腫において、発生頻度や予後との関連が報告されている。この後ろ向き研究の目的は、非小細胞肺癌切除例における予後とABO血液型の関連を検討することである。

**【方法】:**

本研究の対象は333例（男性218例、女性115例）の非小細胞肺癌切除例で、年齢、性別、喫煙状況、術前血清CEA値、術式、腫瘍の組織型、病理病期、術後補助療法、およびABO血液型と予後との関連を検討した。

**【結果】:**

ABO血液型別の5年全生存率（Overall survival: OS）と5年無再発生存率（Disease free survival: DFS）は、それぞれO型83%、71.6%、A型67.2%、62.3%、B型68.8%、68.8%、AB型69.2%、65.3%であった。OSに対する多変量解析で、年齢、性別、喫煙状況、病理病期、術前血清CEA値と同様にABO血液型は独立した予後因子であった（A型 vs. O型のハザード比：2.47、AB型 vs. O型のハザード比：3.62）。DFSに対する多変量解析においても、ABO血液型は独立した予後因子であった。

**【結論】:**

ABO血液型は非小細胞肺癌切除例における独立した予後因子である。ABO血液型と非小細胞肺癌切除例の予後との関連を検討するために、今後外部コホートや大規模な集団での検討が望まれる。

## INTRODUCTION

Lung cancer is the leading cause of cancer death worldwide. In 2008, 1.6 million people received a new diagnosis of lung cancer, comprising 13% of all new cancer diagnoses, and 1.37 million people died of lung cancer, accounting for 18% of all cancer deaths in the world.^1^^,^^2^ Patients with lung cancer, especially non-small cell lung cancer (NSCLC) without metastatic disease, are considered to be candidates for surgical resection. Although complete resection is often achieved in such patients, some patients experience relapse after surgery. In order to improve the outcomes of surgically managed patients, new prognostic factors must be explored, in addition to established factors such as a high preoperative or postoperative serum carcinoembryonic antigen (CEA) level,^3^^,^^4^ positive results on pleural lavage cytology,^5^ and a high standardized uptake value on positron emission tomography.^6^

At the beginning of the 20th century, the Austrian scientist Karl Landsteiner identified the ABO blood group system. This discovery was the first detection of a genetic polymorphism in humans. Recently, an increasing number of studies have shown that the ABO blood group, in addition to its fundamental role in transfusion medicine, plays an important role in several human diseases, including venous thromboembolism (VTE),^7^ coronary heart disease,^8^ ischemic stroke,^9^ and neoplastic disorders. Some reports have evaluated the association between the ABO blood group and the incidence of various types of malignancies, including gastric cancer,^10^ pancreatic cancer,^11^ and renal cell carcinoma.^12^ In addition, there are two studies that evaluated the association between the ABO blood group and the prognosis of cancer patients, such as those with pancreatic cancer^13^ and renal cell carcinoma.^14^ Both studies found that the prognosis of blood group O patients is superior to that of non-blood group O patients.^13^^,^^14^ However, few studies have assessed the relationship between the ABO blood group and the prognosis among patients with lung cancer. The aim of the present study was to clarify the prognostic significance of the ABO blood group in patients with resected NSCLC.

## METHODS

Between January 2004 and December 2007, 337 patients with NSCLC underwent pulmonary resections at Nagoya University Hospital. In order to evaluate both overall survival (OS) and disease-free survival (DFS), 4 patients who had pleural dissemination at the thoracotomy (R2 resection: macroscopic residual tumor) were excluded from this study. All of the eligible patients underwent an ABO blood group examination prior to surgery. The ABO blood group evaluations were carried out via agglutination technology using the **^®^**BioVue system (Ortho Clinical Diagnostics Japan, Tokyo, Japan). All clinical and pathological data were collected using a clinical database and charts. In addition to the ABO blood group, examined factors included age, sex, smoking status, preoperative serum CEA level, operative procedures, histology, postoperative adjuvant therapy, and pathological stage (p-stage).

Fisher’s exact test and an analysis of variance were used to compare each variable between the blood groups, as appropriate. OS was calculated from the date of surgery to death. DFS was defined as the period from the date of surgery to the date of identification of recurrent disease or death from any cause. Two patients were excluded from DFS analysis because of missing data. The Kaplan-Meier method was used to calculate the survival rate with a 95% confidence interval (CI), and the log-rank test was used to compare the survival curves. A Cox proportional hazard model was used for the univariate and multivariate survival analyses. Reported *P* values were two-sided, and those less than 0.05 were considered statistically significant. The statistical analyses were performed using the computer software program STATA/SE Ver.12.1 (State Corp., College Station, TX, USA). The Institutional Review Board of Nagoya University Hospital approved this retrospective study.

## RESULTS

The patient characteristics are shown in Table 1. This study included 218 men and 115 women, ranging in age from 31 to 85 years (median: 68 years). The median observation period in the survivors was 73 months (range: 1–107 months). The pathological characteristics were as follows: 210 tumors were adenocarcinomas (ADs), 93 tumors were squamous cell carcinomas (SQs), and 30 tumors were other NSCLCs (others). Meanwhile, 227 patients had p-stage I disease, 49 had p-stage II disease, and 57 had p-stage III disease. Sixty-eight patients (20.4%) received adjuvant therapy (chemotherapy and/or radiation therapy) after surgery.

**Table 1.** Patient characteristics

		All	Blood group								

			Group O		Group A		Group B		Group AB		*P* value
		*n* = 333	*n* = 108 (32.4%)	%	*n* = 140 (42.1%)	%	*n* = 59 (17.7%)	%	*n* = 26 (7.8%)	%	
Age, median (range)		68 (31–85)	68 (40–84)		68 (31–85)		67 (40–85)		71 (40–83)		0.511
Sex	Female	115	38	35.2	55	39.3	13	22	9	34.6	0.128
	Male	218	70	64.9	85	60.7	46	78	17	65.4	
Smoking status	Never	93	31	28.7	43	30.7	13	22	6	23.1	0.585
	Ever/Current	237	76	70.4	95	67.9	46	78	20	76.9	
	Unknown	3	1	0.9	2	1.4	0		0		
CEA (ng/mL)	≤5	219	73	67.6	90	64.3	39	66.1	17	65.4	0.986
	>5	103	34	31.5	41	29.3	19	32.2	9	34.6	
	Unknown	11	1	0.9	9	6.4	1	1.7	0		
Operative procedure	Wedge/Seg	37	6	5.4	25	17.9	5	8.5	1	3.8	0.044
	Lob	276	97	90	107	76.4	49	83	23	88.5	
	Pn	20	5	4.6	8	5.7	5	8.5	2	7.7	
Histology	AD	210	60	55.6	94	67.1	37	62.7	19	73.1	0.353
	SQ	93	34	31.5	34	24.3	19	32.2	6	23.1	
	Others	30	14	12.9	12	8.6	3	5.1	1	3.8	
p-stage	I	227	72	66.7	98	70	37	62.7	20	77	0.037
	II	49	10	9.3	20	14.3	16	27.1	3	11.5	
	III	57	26	24	22	15.7	6	10.2	3	11.5	
Adjuvant therapy	No	265	81	75	113	80.7	52	88.1	19	73.1	0.17
	Yes	68	27	25	27	19.3	7	11.9	7	26.9	

AD, adenocarcinoma; CEA, carcinoembryonic antigen; Lob, lobectomy; Pn, pneumonectomy; p-stage, pathological stage; Seg, segmentectomy; SQ, squamous cell carcinoma; Wedge, wedge resection.

**Figure 1.**
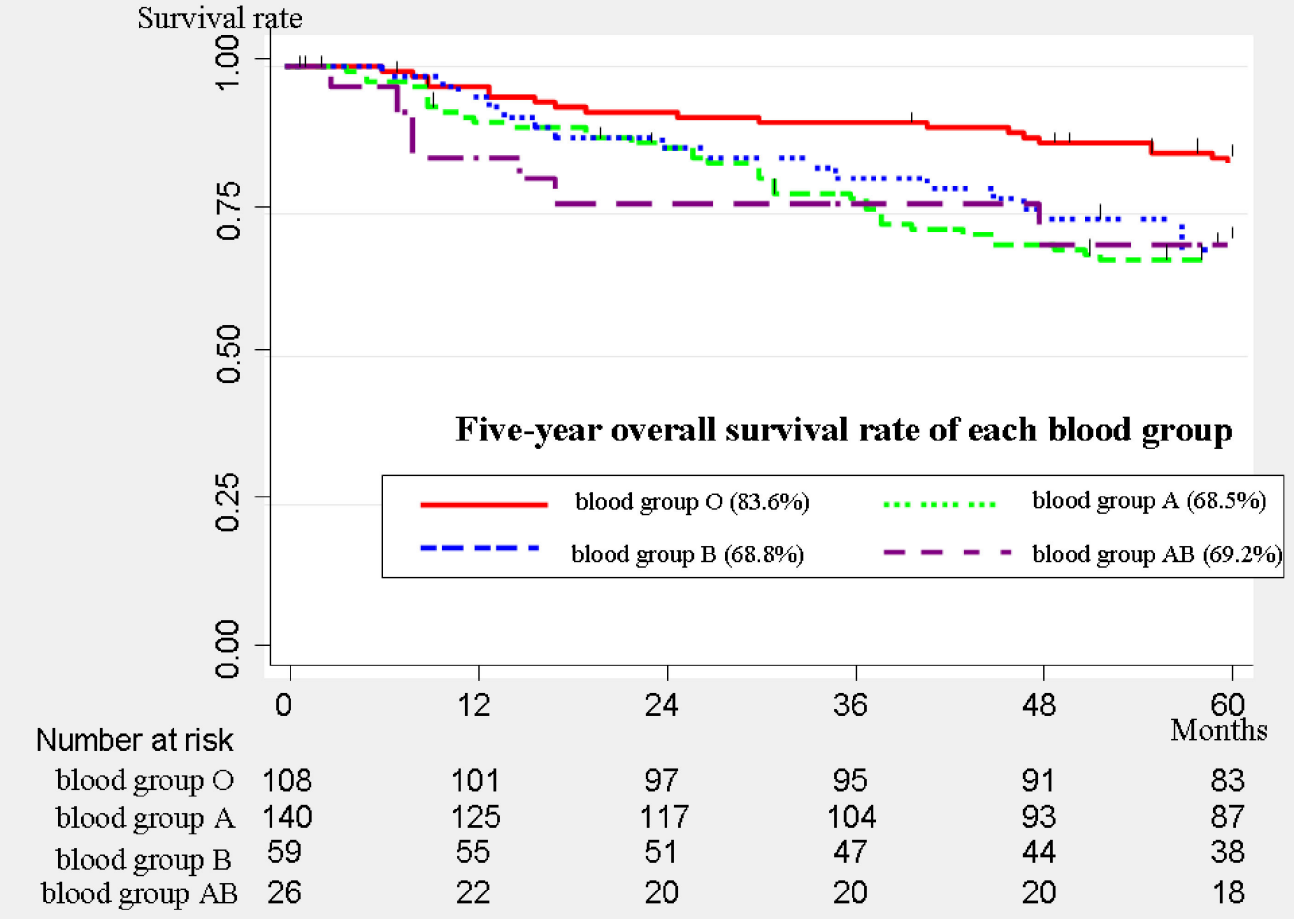
Overall survival curves of all patients (*n* = 333). The 5-year overall survival rate was 83.6% (95% confidence interval [CI], 75%–89.5%) for blood group O, 68.5% (95% CI, 59.8%–75.6%) for blood group A, 68.8% (95% CI, 55.2%–79.1%) for blood group B, and 69.2% (95% CI, 47.8%–83.3%) for blood group AB. The patients in blood group O showed significantly better survival than the patients in the non-O blood groups (*P* = 0.016).

There were 108 (32.4%) patients with blood group O, 140 (42.1%) with blood group A, 59 (17.7%) with blood group B, and 26 (7.8%) with blood group AB. The distribution of each blood group was similar to that of the general population in Japan.^15^ There were significantly more advanced-stage patients in blood group O than in any other group (*P* = 0.037). The overall survival curves of each blood group are shown in Figure 1. The five-year overall survival (OS) rate was 83.6% (95% CI, 75.0%–89.5%) for blood group O, 68.5% (95% CI, 59.8%–75.6%) for blood group A, 68.8% (95% CI, 55.2%–79.1%) for blood group B, and 69.2% (95% CI, 47.8%–83.3%) for blood group AB. The patients in blood group O showed significantly better survival than the patients in non-O blood groups (*P* = 0.016). Stratified analysis by p-stage revealed that the association between the ABO blood group and overall survival was similar in each p-stage group.

A univariate analysis for OS showed that age (per 1 year: hazard ratio [HR] 1.05), sex (male vs. female: HR 2.71), smoking status (ever/current vs. never: HR 1.16), preoperative serum CEA level (>5 vs. ≤5: HR 2.56), histology (SQ vs. AD: HR 2.3, others vs. AD: HR 2.16), p-stage (stage II vs. stage I: HR 2.12, stage III vs. stage I: HR 2.99), adjuvant therapy (Yes vs. No: HR 0.56), and blood group (group A vs. group O: HR 1.88) were significant prognostic factors (Table 2). Multivariate analysis for OS showed that age (per 1 year: HR 1.05), sex (male vs. female: HR 2.28), smoking status (ever/current vs. never: HR 1.26), p-stage (stage II vs. stage I: HR 2.21, stage III vs. stage I: HR 5.78), adjuvant therapy (Yes vs. No: HR 0.45), and blood group (group A vs. group O: HR 2.47; *P* = 0.001, group AB vs. group O: HR 3.62; *P* = 0.002) were independent significant prognostic factors (Table 2). Blood groups A and AB, which express the blood group A antigen on erythrocytes, were independently associated with poor OS among patients with resected NSCLC.

**Table 2.** Univariate and multivariate analysis for overall survival

		Univariate analysis				Multivariate analysis			
	
		HR	95% CI		*P* value	HR	95% CI		*P* value
Age (/year)		1.05	1.02	1.07	<0.001	1.05	1.02	1.08	<0.001
Sex	Female	reference				reference			
	Male	2.71	1.64	4.46	<0.001	2.28	1.34	3.88	0.002
Smoking status	Never	reference				reference			
	Ever/Current	1.16	1.01	1.32	0.03	1.26	1.02	1.55	0.029
CEA (ng/mL)	≤5	reference				reference			
	>5	2.56	1.71	3.82	<0.001	1.09	0.99	1.19	0.052
Operative procedure	Wedge/Seg	reference				reference			
	Lob	0.69	0.39	1.19	0.185	0.69	0.38	1.26	0.224
	Pn	1.1	0.47	2.6	0.827	0.68	0.25	1.91	0.47
Histology	AD	reference				reference			
	SQ	2.3	1.52	3.49	<0.001	1.55	0.95	2.53	0.08
	Others	2.16	1.15	4.07	0.017	1.98	0.99	3.91	0.05
p-stage	I	reference				reference			
	II	2.12	1.27	3.56	0.004	2.21	1.26	3.89	0.006
	III	2.99	1.9	4.72	<0.001	5.78	3.42	9.76	<0.001
Adjuvant therapy	No	reference				reference			
	Yes	0.56	0.32	0.98	0.031	0.45	0.24	0.82	0.01
Blood group	O	reference				reference			
	A	1.88	1.15	3.08	0.012	2.47	1.45	4.21	0.001
	B	1.55	0.84	2.87	0.164	1.47	0.77	2.79	0.244
	AB	1.98	0.94	4.17	0.071	3.62	1.61	8.15	0.002

AD, adenocarcinoma; CEA, carcinoembryonic antigen; CI, confidence interval; HR, hazard ratio; Lob, lobectomy; Pn, pneumonectomy; p-stage, pathological stage; Seg, segmentectomy; SQ, squamous cell carcinoma; Wedge, wedge resection.

**Figure 2.**
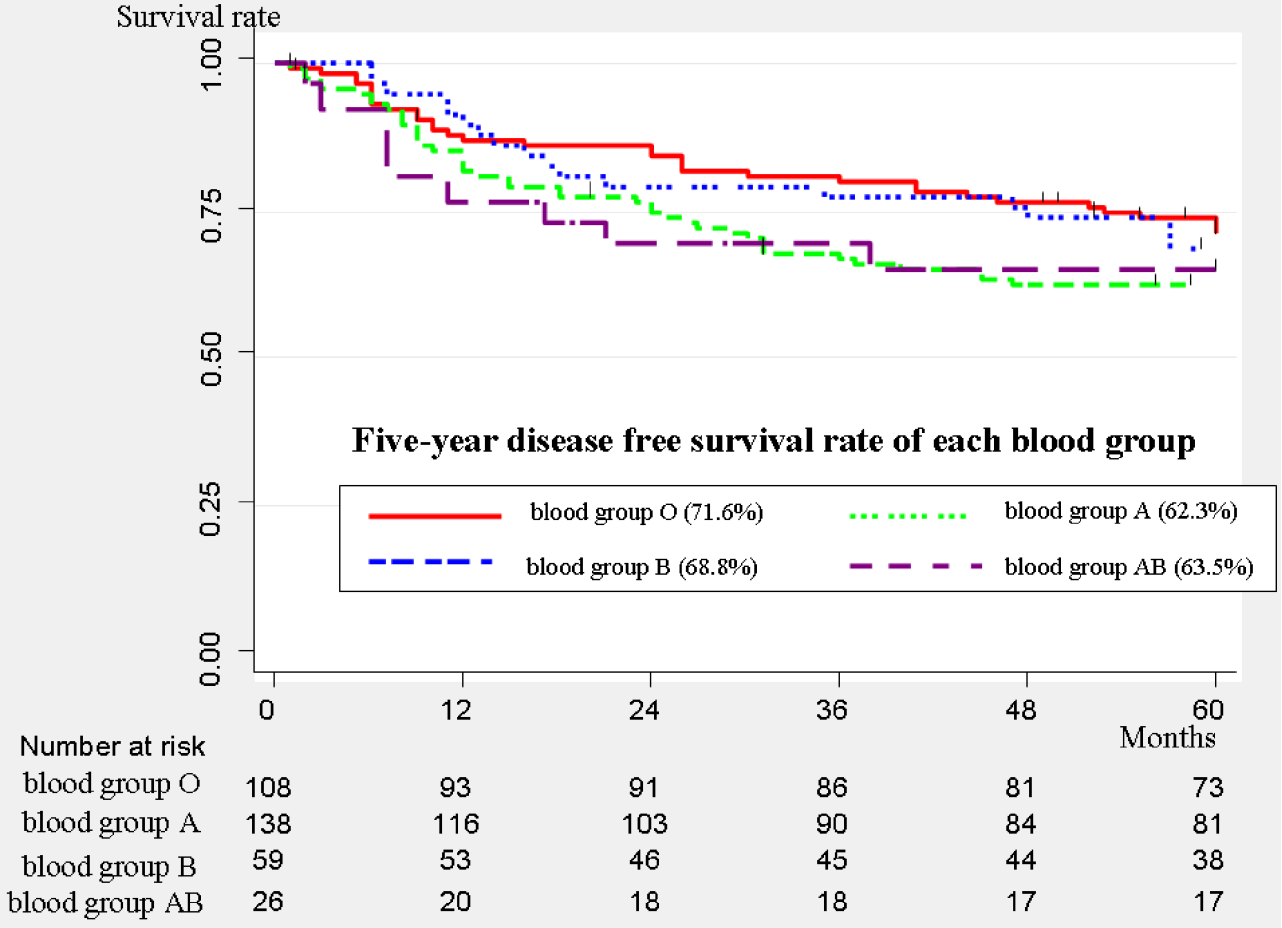
Disease-free survival curves of all patients (*n* = 331). The 5-year disease-free survival rate was 71.6% (95% confidence interval [CI], 61.9%–79.2%) for blood group O, 62.3% (95% CI, 53.5%–69.8%) for blood group A, 68.8% (95% CI, 55.2%–79.1%) for blood group B, and 65.3% (95% CI, 44%–80.2%) for blood group AB.

The DFS curves of each blood group are shown in Figure 2. The 5-year DFS rate was 71.6% (95% CI, 61.9%–79.2%) for blood group O, 62.3% (95% CI, 53.5%–69.8%) for blood group A, 68.8% (95% CI, 55.2%–79.1%) for blood group B, and 65.3% (95% CI, 44%–80.2%) for blood group AB.

Univariate analysis for DFS showed that age, sex, preoperative serum CEA level, histology, and p-stage were significant prognostic factors (Table 3). Multivariate analysis for DFS showed that age (per 1 year: HR 1.04), sex (male vs. female: HR 1.87), p-stage (stage II vs. stage I: HR 2.1, stage III vs. stage I: HR 6.08), and blood group (group A vs. group O: HR 1.78, *P* = 0.014; group AB vs. group O: HR 2.49, *P* = 0.016) were independent significant prognostic factors (Table 3). Similar to the results of multivariate OS analysis, blood groups A and AB were independently associated with a poor DFS among patients with resected NSCLC.

**Table 3.** Univariate and multivariate analysis for disease-free survival

		Univariate analysis				Multivariate analysis			
	
		HR	95% CI		*P* value	HR	95% CI		*P* value
Age (/year)		1.03	1.01	1.06	0.002	1.04	1.01	1.06	0.002
Sex	Female	reference				reference			
	Male	2.28	1.47	3.55	<0.001	1.87	1.16	3	0.01
Smoking status	Never	reference				reference			
	Ever/Current	1.12	0.99	1.28	0.073	1.19	0.97	1.44	0.09
CEA (ng/mL)	≤5	reference				reference			
	>5	1.1	1.01	1.19	0.028	1.09	0.99	1.19	0.055
Operative procedure	Wedge/Seg	reference				reference			
	Lob	0.73	0.44	1.23	0.234	0.63	0.36	1.11	0.114
	Pn	1.45	0.68	3.09	0.339	0.63	0.25	1.56	0.317
Histology	AD	reference				reference			
	SQ	1.91	1.29	2.8	0.001	1.24	0.78	1.95	0.364
	Others	1.89	1.04	3.45	0.037	1.57	0.83	2.98	0.164
p-stage	I	reference				reference			
	II	1.91	1.18	3.19	0.009	2.1	1.22	3.61	0.007
	III	4.15	2.76	6.25	<0.001	6.08	3.78	9.78	<0.001
Adjuvant therapy	No	reference				reference			
	Yes	0.97	0.62	1.52	0.897	0.73	0.45	1.21	0.222
Blood group	O	reference				reference			
	A	1.44	0.94	2.21	0.097	1.78	1.12	2.84	0.014
	B	1.07	0.61	1.88	0.817	1.09	0.6	1.97	0.776
	AB	1.52	0.77	3	0.231	2.49	1.18	5.23	0.016

AD, adenocarcinoma; CEA, carcinoembryonic antigen; CI, confidence interval; HR, hazard ratio; Lob, lobectomy; Pn, pneumonectomy; p-stage, pathological stage; Seg, segmentectomy; SQ, squamous cell carcinoma; Wedge, wedge resection.

## DISCUSSION

Recently, several studies have suggested important roles for the ABO blood group in the development of hemostasis and neoplastic disease, as ABO antigens are highly expressed on the surface of a variety of human cells and tissues.^16^ As listed in Table 4, there are a number of reports regarding the relationship between the ABO blood group and the incidence of several types of cancers. Affirmation of this relationship has been reported by some studies of renal cell carcinoma,^12^ extrahepatic cholangiocarcinoma,^17^ nasopharyngeal carcinoma,^18^ ovarian cancer,^19^^–^^21^ breast cancer,^22^ gastric cancer,^10^^,^^23^^,^^24^ pancreatic cancer,^11^^,^^25^^,^^26^ and lung cancer.^27^ Meanwhile, no affirmation of the relationship has been reported for colorectal cancer^28^ and cervical/endometrial cancer.^20^ Trends in the relationship between blood group and incidence of various types of cancers have been noted; namely, blood groups O or non-A show a low incidence of cancer, while blood groups non-O or A demonstrate a higher incidence of cancer. Among these studies, the relationship between the ABO blood group and the incidence of gastric and/or pancreatic cancer is considered to be reliable and convincing, as the studies were large-scale meta-analyses.^10^^,^^11^

**Table 4.** Previously reported studies on the relationship between the ABO blood group and the incidence of malignant tumors

First author	Reference number	Year	Country	Type of cancer	Type of study	Results	Blood group	

							with low incidence	with high incidence
Joh	12	2012	USA	renal cell carcinoma	prospective cohort study	positive	O	non-O
Zhou	17	2013	China	extrahepatic cholangiocarcinoma	case-control study	positive	O	A
Sheng	18	2013	China	nasopharyngeal carcinoma	case-control study	positive	O	A, AB
Gates	19	2011	USA	ovarian cancer	prospective cohort study	positive	non-B	B
Yuzhalin	20	2012	Russia	ovarian cancer	case-control study	positive	O	premenopausal in A, postmenopausal in B and AB
Poole	21	2012	USA	ovarian cancer	meta analysis	positive	O	A
Miao	22	2013	China	breast cancer	meta analysis	positive	O	A
Edgren	23	2010	Sweden	gastric cancer	population-based cohort study	positive	O	A
Nakao	24	2011	Japan	gastric cancer	case-control study	positive	non-A	A
Wang	10	2012	China	gastric cancer	meta analysis	positive	non-A	A
Wolpin	25	2009	USA	pancreatic cancer	prospective cohort study	positive	O	non-O
Nakao	26	2010	Japan	pancreatic cancer	case-control study	positive	O	non-O
Risch	11	2012	USA	pancreatic cancer	meta analysis	positive	O	A
Urun	27	2013	Turkey	lung cancer	case-control study	positive	O	non-O
Khalili	28	2011	USA	colorectal cancer	prospective cohort study	negative		
Yuzhalin	19	2012	Russia	cervical and endometrial cancer	case-control study	negative		

There are also a few studies regarding the relationship between the ABO blood group and the prognosis in patients with malignant tumors. Kaffenberger et al reported that the non-O blood type was found to be associated with a significantly decreased OS among 900 surgically managed patients with renal cell carcinoma according to a multivariate survival analysis (HR 1.68; 95% CI, 1.18–2.39).^14^ The authors also reported that the non-O blood type was associated with marginally decreased DFS in the same cohort (HR 1.53; 95% CI, 0.97–2.41). Rahbari et al analyzed a total of 627 patients who underwent resection for pancreatic ductal adenocarcinoma and revealed a favorable and independent impact of blood group O (vs. non-O) on OS according to a multivariate survival analysis (HR 0.78; 95% CI, 0.62–0.99).^13^ Furthermore, OuYang et al recently demonstrated the prognostic value of the ABO blood group in patients with nasopharyngeal carcinoma treated with intensity-modulated radiotherapy (IMRT) or conventional radiotherapy (CRT).^29^ In their multivariate survival analysis, patients with blood type A had a significantly lower OS and distant metastasis-free survival than those in the non-A group, among both the IMRT group (*n* = 924) and CRT group (*n* = 1193). These results are consistent with our observations, in which we found that the OS and DFS of the resected NSCLC patients in blood group A or AB are significantly poorer than that of patients in blood group O, despite the higher incidence of advanced cancer among blood group O individuals.

The mechanisms by which the ABO blood group influences the prognosis of cancer patients have not been fully investigated. The A and B antigens are expressed on the surface of red blood cells as well as numerous other tissues throughout the body,^30^ including lung cancer tissues.^31^ One hypothesis is that ABO antigens in tumor cells play an important role in intracellular adhesion and membrane signaling, both of which are critical to the progression and spread of malignant cells.^32^ Lee et al reported that the expression of blood group antigen A on lung cancer tissue is an important favorable prognostic factor in blood group A patients.^31^ We speculate that the A and B antibodies in the plasma of blood group O patients have protective effects against tumor cell progression.

Recently, two genome-wide association studies have suggested that single nucleotide polymorphisms at the ABO gene locus are associated with two serum markers, namely tumor necrosis factor-α (TNF-α) and soluble intracellular adhesion molecule-1 (sICAM-1).^33^^,^^34^ TNF-α is an inflammatory cytokine that affects tumor progression. In addition, the levels of sICAM-1 are known to be elevated in several types of malignancies and may play a role in escape from immune surveillance by tumor cells.^35^ Therefore, we also suspect that the ABO gene locus influences the prognosis of cancer patients via the effects of these serum proteins.

There are some limitations to our retrospective analysis. First, the number of study subjects was small; however, to our knowledge, this is the first report to show the prognostic significance of the ABO blood type in surgically managed NSCLC patients. Secondly, our data regarding the ABO blood groups were obtained using a serological technique based on the phenotype, not the genotype, of the blood group. Nakao et al reported that the number of non-O alleles was found to be associated with an increased risk of pancreatic cancer in a Japanese population.^26^ The number of non-O alleles may therefore have an additive effect on the prognosis of patients with NSCLC.

### Conclusion

Our multivariate survival analysis showed the ABO blood group to be an independent prognostic factor in addition to age, sex, smoking status, p-stage, and serum CEA level. The blood group A antigen may have a negative effect on the prognosis of surgically managed patients with NSCLC. Studies using other larger cohorts are needed to confirm a robust relationship between ABO blood group and the prognosis of patients with resected NSCLC.

## ONLINE ONLY MATERIAL

Abstract in Japanese.
