# Supplementary material for: The ABO Blood Group is an Independent Prognostic Factor in Patients With Resected Non-small Cell Lung Cancer
Source: J Epidemiol. 2015 Feb 5;25(2):110–6. doi: 10.2188/jea.JE20140102 (PMC4310871; doi:10.2188/jea.JE20140102)
Supplement: Abstract in Japanese. [file je-25-110-s001.pdf]

ABO 血液型は非小細胞肺癌切除例における独立した予後因子である

福本紘一、谷口哲郎、宇佐美範恭、川口晃司、福井高幸、石黒太志、中村彰太、横井香平
名古屋大学大学院医学系研究科病態外科学講座　呼吸器外科学

【背景】ABO 血液型はいくつかのがん腫において、発生頻度や予後との関連が報告されている。この後ろ向き研究の目的は、非小細胞肺癌切除例における予後と ABO 血液型の関連を検討することである。

【方法】本研究の対象は 333 例（男性 218 例、女性 115 例）の非小細胞肺癌切除例で、年齢、性別、喫煙状況、術前血清 CEA 値、術式、腫瘍の組織型、病理病期、術後補助療法、および ABO 血液型と予後との関連を検討した。

【結果】ABO 血液型別の 5 年全生存率（Overall survival: OS）と 5 年無再発生存率（Disease free survival: DFS）は、それぞれ O 型 83%、71.6%、A 型 67.2%、62.3%、B 型 68.8%、68.8%、AB 型 69.2%、65.3%であった。OS に対する多変量解析で、年齢、性別、喫煙状況、病理病期、術前血清 CEA 値と同様に ABO 血液型は独立した予後因子であった（A 型 vs. O 型のハザード比：2.47、AB 型 vs. O 型のハザード比：3.62）。DFS に対する多変量解析においても、ABO 血液型は独立した予後因子であった。

【結論】ABO 血液型は非小細胞肺癌切除例における独立した予後因子である。ABO 血液型と非小細胞肺癌切除例の予後との関連を検討するために、今後外部コホートや大規模な集団での検討が望まれる。

キーワード：非小細胞肺癌、ABO 血液型、手術、予後因子
